# Magnetically Enhanced Liquid SERS for Ultrasensitive Analysis of Bacterial and SARS-CoV-2 Biomarkers

**DOI:** 10.3389/fbioe.2021.735711

**Published:** 2021-09-29

**Authors:** Zhang Ji, Chuan Zhang, Yang Ye, Jiali Ji, Hongguang Dong, Erik Forsberg, Xiaoyu Cheng, Sailing He

**Affiliations:** ^1^ National Engineering Research Center for Optical Instruments, College of Optical Science and Engineering, Zhejiang University, Hangzhou, China; ^2^ Ningbo Research Institute, Ningbo, China; ^3^ ZJU-TU/e Joint Research Institute of Design, Optoelectronic and Sensing, Hangzhou, China

**Keywords:** SERS, COVID-19, SARS-CoV-2, bacteria, analysis

## Abstract

In this work, it is shown that surface-enhanced Raman scattering (SERS) measurements can be performed using liquid platforms to perform bioanalysis at sub-pM concentrations. Using magnetic enrichment with gold-coated magnetic nanoparticles, the high sensitivity was verified with nucleic acid and protein targets. The former was performed with a DNA fragment associated with the bacteria *Staphylococcus aureus*, and the latter using *IgG* antibody, a biomarker for COVID-19 screening. It is anticipated that this work will inspire studies on ultrasensitive SERS analyzers suitable for large-scale applications, which is particularly important for *in vitro* diagnostics and environmental studies.

## Introduction

Surface-enhanced Raman scattering (SERS) spectroscopy holds promises for ultrasensitive detection of analytes in complex environments (Ding et al., 2016; Langer et al., 2020). This is of significance for two reasons: First, disease-related biomarkers are often present at concentrations lower than the limit of detection (LoD) of existing techniques (Wu et al., 2019). Examples of such samples include circulating tumor biomarkers (e.g., microRNA and cells), proteins, exosomes, bacteria pathogens, and SARS-CoV-2 biomarkers in blood (Mattioli et al., 2020; Jiang et al., 2020a; Sitjar et al., 2021). Second, SERS is capable of detecting single molecules (Bell et al., 2020; Zheng et al., 2015; Kneipp et al., 2006), and as a vibrational spectroscopic tool, the Raman spectra can provide molecular fingerprints of the sample that is highly specific (Zheng et al., 2013; Zheng et al., 2015; Ye et al., 2019; Langer et al., 2020; Mattioli et al., 2020), the signal of which is significantly enhanced when performed in ultraviolet (Renard et al., 2019; Chen et al., 2021). The dual sensitivity/selectivity of SERS is desirable for several applications, particularly *in vitro* diagnostics and environmental monitoring (Long and Gooding, 2016; Liu et al., 2017; Wei et al., 2017; Zhang et al., 2018; Chen et al., 2020a).

One goal for SERS is ultrasensitive quantification of rare species (e.g., sub-pM) using techniques that are both accessible and reproducible (Joseph et al., 2012). Conventional SERS measurements typically require substrates made with, for example, electron beam lithography, self-assembled nanoparticle arrays on solid support, or simply mixing analytes with nanoparticle suspensions (Ciallam et al., 2012; Thai et al., 2012). Among them, nanofabrication produces well-defined substrates but is challenging to manufacture at large scale (Ying et al., 2008). Colloidal suspensions are attractive for their straightforward production, but the resulting organization of the substrate can be poor due to the random structures formed (Banholzer et al., 2008). Self-assembled super-lattice arrays on solid surfaces are promising for their reasonably good organization and easy fabrication (Thai et al., 2012; Ye et al., 2019). Issues remain in developing SERS-based detection to address real-world problems, where factors such as sensitivity, specificity, cost of the instrument, and reproducibility of the assay should all be addressed to meet production requirements and analytical standards (Fan et al., 2020). Of interest is recent studies showing that well-defined SERS substrates can be produced directly in the liquid-state *via* self-assembling gold nanoparticles between the phases of immiscible solvents (Ma et al., 2016; Tian et al., 2018; Du et al., 2019; Su et al., 2019). This is attractive as gold colloids can be obtained easily, and it provided a simple and reproducible method to perform SERS at minimal cost. This was suitable for large-scale applications which predominantly rely on solution-based measurements.

In this work, we demonstrate that the limit of detection (LoD) of liquid interfacial SERS can be improved by three orders of magnitude to allow pM-level (parts per trillion) immunosensing. We show that using surface-modified, gold-coated magnetic nanoparticles (Au@MNPs), nucleic acid and protein targets with concentration lower than that of pM could be quantified. Protein assays were performed using *IgG* antibodies, a biomarker elevated in patients infected with SARS-CoV-2 viruses (Yao et al., 2020). The results were compared with paper-based lateral flow assays which are currently used for COVID-19 antibody screening (Chan et al., 2020). The experimental results were supported with a simple electromagnetic (EM) simulation, showing significantly enhanced SERS signals in the gap formed between the Au@MNP and the substrate. The current work demonstrated that magnetically enhanced liquid SERS could be used to detect analytes of ultralow abundance, which is pertinent for clinical and environmental applications, such as *in vitro* diagnostics and water quality monitoring (Beveridge et al., 2011).

## Results and Discussions

### Analysis of *Staphylococcus aureus* Nucleic Acid

**FIGURE 1:**
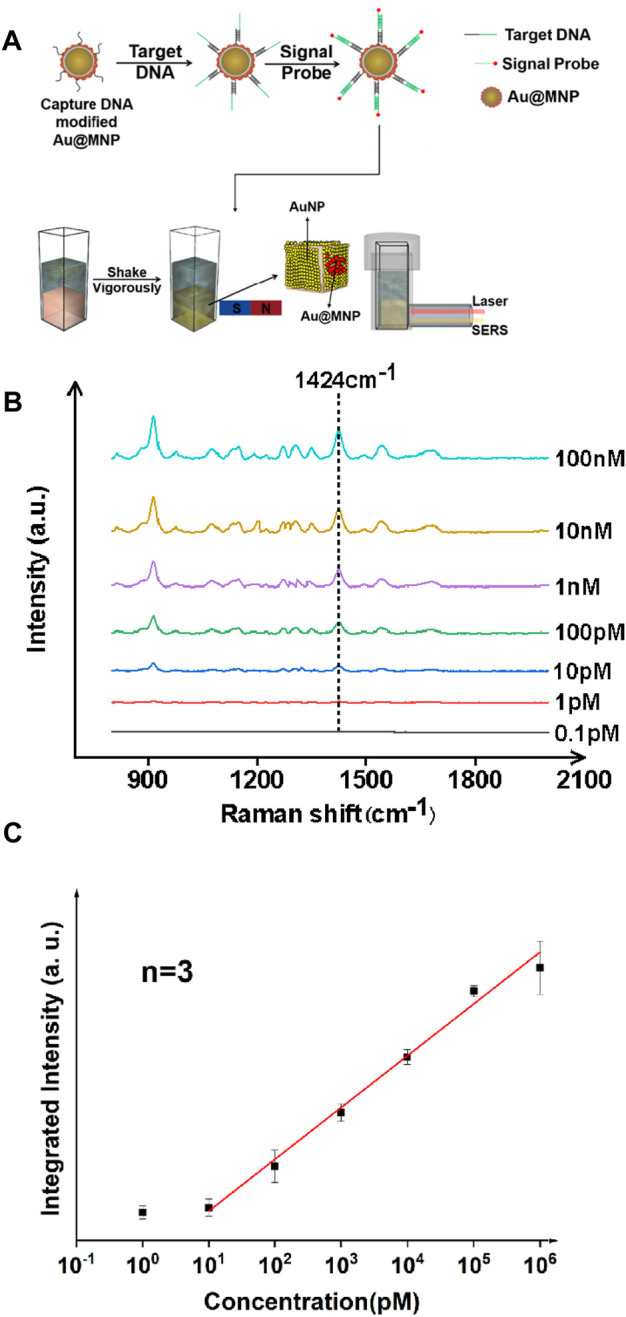
Ultrasensitive detection of nucleic acid fragments of *S. aureus* with the liquid-state SERS platform. **(A)** Sample preparation and analysis flowchart. **(B)** SERS spectra of nucleic acid samples with different concentrations. **(C)** Concentration *vs.* integrated intensity curve using a signature peak at 1,424 cm^−1^, showing linearity of the SERS signal from ∼10 pM to 10^6^ pM. The error bar was obtained by finding mean to six measurements on the same sample, and averaged out of three samples. The signal probe used in this case was Cy3, a standard Raman dye for DNA labeling.

The synthesis and characterization of gold-coated magnetic nanoparticles is discussed in detail in the supporting information. In short, Au@MNPs were prepared *via* typical solution-based approaches *via* electrostatic adsorption, and then coated with DNA to form well-organized self-assembled monolayers on the surface. The performance of magnetically enhanced liquid SERS was first tested for a nucleic acid target. In this work, *Staphylococcus aureus* (*S. aureus*) was chosen, as it played a critical role in cross-infections among hospitalized patients (Pazos-Perez et al., 2016). A three-fragment assay was performed (Figure 1A), in which single-stranded DNA (surface strand, 20 bases) was attached onto Au@MNPs *via* gold–thiol bonding using freeze-induced surface modifications (Liu and Liu, 2019). The Au@MNPs were then coated with polyethylene glycol to reduce nonspecific adsorptions, after which target strands (*S. aureus* DNA fragments, 40 bases) were added. The sticky end of the target DNA was complementary to the signal probe strand labeled with Cy3 (20 bases), a standard dye for SERS analysis. In this way, thermodynamically stable, double-stranded surface geometry can be formed when the target strand was present.

The SERS spectra were measured for targets at various concentrations, as shown in Figure 1B. Analysis was performed in solution when incubating Au@MNPs with the target strands prior to applying the magnetic field. Quantification was performed using an excitation source of 785 nm continuous wave laser (power: 100 mW, integration time: 15 s) and signal recorded with a Raman fiber spectrometer. Using a signature peak of the Cy3 dye at 1,424 cm^−1^, which corresponds to the C-H in-plane deformation mode (Li et al., 2020a), a linear trend between the concentration of the nucleic acid and peak intensity was observed in the dynamic range between 10 pM and 10^6^ pM, with a linear correlation coefficient of >0.99. A limit of detection was found to be ∼8.5 pM using a signal-to-noise (S/N) ratio above three. The use of the liquid SERS substrate was important because it was found that the Raman signature was weak with only using Au@MNPs (Supplementary Figure S1). This also suggested that the liquid-state SERS substrate remained stable even when magnetic nanoparticles were attached to it.

**FIGURE 2:**
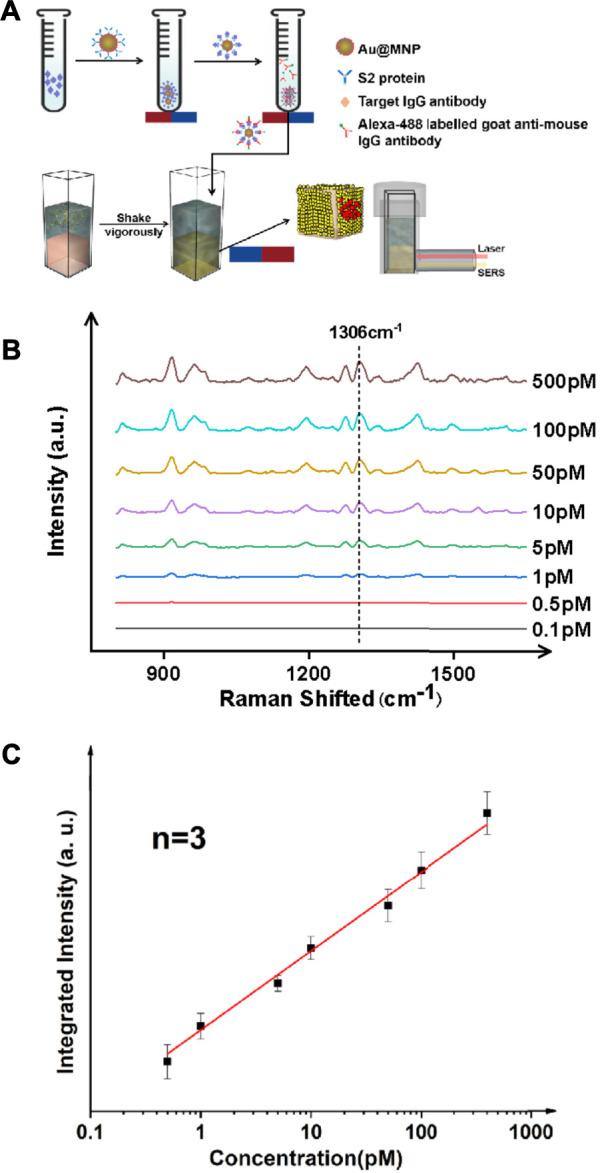
Ultrasensitive detection of protein targets (IgG antibody) in a typical sandwich assay format. **(A)** Schematic illustration of the antibody detection process. The antigen here is the IgG antibody which is elevated in the serum of patients with COVID-19. An S2 protein fragment was used as the primary antibody, and an Alexa 488 dye labeled anti-IgG antibody was used as the secondary antibody. **(B)** SERS spectra for targets of various concentrations. **(C)** Linear trend showing a detection limit of IgG down to ∼1 pM.

We further verified the performance of the magnetically enhanced SERS with protein targets, as seen in Figure 2. The SARS-CoV-2 antibody, immunoglobulin G (*IgG*), was chosen as the target antigen. The *IgG* antibody binds strongly to the *S* spike protein fragments on the surface of SARS-Cov-2 virus, and is a standard biomarker for tracking and screening COVID-19 patients, typically using blood samples (Chen et al., 2020b; Yao et al., 2020). In practice, the concentration of *IgG* in serum is often below the LoD of many of the existing technologies, down to the pM range (Li et al., 2020b; Kohmer et al., 2020). This is challenging for early-stage/asymptomatic infections. Given the scale and severity of the COVID-19 pandemic with a possibility to prevail in the next few years given the many emerging mutants, such as Delta and Lamda variants (Kemp et al., 2021), it is of importance to develop reproducible methods to quantify *IgG* antibodies with high sensitivity (Deeks et al., 2020).

**FIGURE 3:**
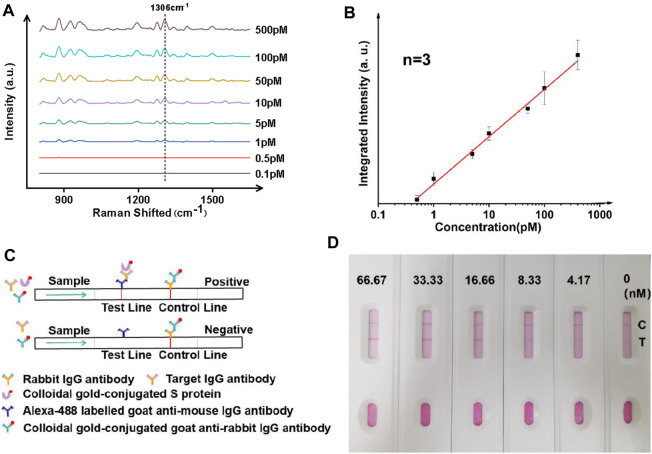
Ultrasensitive analysis of IgG antibodies using whole blood mimic and comparison to a paper-based lateral flow assay. **(A)** Typical SERS spectra with targets of different concentrations. **(B)** Linearity showing increasing intensity of the signature peak at varied concentrations of the analyte. Three samples were used for each measurement. **(C)** Principle and **(D)** results of the paper-based lateral flow sensor, showing a LoD of between 4 and 8 nM in a typical paper-based lateral flow assay.

Au@MNPs were modified with a biotinylated PEG layer before being coated with a streptavidin-labeled, anti-*IgG* primary antibody, as shown in Figure 3A. Using a secondary antibody (Alexa-488 labeled goat anti-mouse antibody) to capture *IgG*, the assay was conducted in a standard sandwich format, and SERS measurements were performed following magnetic pull-down. The reason that Alexa 488 (instead of Cy3 used for DNA measurements) was used was due to the commercial availability of the dye as protein labels. As can be seen in Figure 3, B-C, with target enhancement, clean SERS spectra were obtained for target concentrations below 1 nM. Using a signature peak in Alexa 488, which occurred at 1,306 cm^−1^, corresponding to the amide stretch (Chen et al., 2011), a clear linearity between peak intensity and analyte concentration was seen between ∼0.5 and ∼500 pM, a detection range inaccessible to current liquid-SERS measurements. In this case, an LoD was found to be ∼0.37 pM using the S/N ratio of 3.

Since the majority of *IgG* tests for COVID-19 in clinical environments are performed with serum samples, the performance of the SERS analyzer was further studied using a commercially obtained whole blood mimic. As seen in Figures 3A,B, the performance of the magnetically enhanced SERS sensor was comparable to when using buffer-based systems, with a linear trend observed when concentration of the target was between ∼0.5 and ∼500 pM, with a correlation coefficient over 0.98. An LoD of ∼0.42 pM was determined using the S/N ratio of above three. The fact that the assay could be performed using whole blood mimic was promising, as the use of the PEG layer on the surface of nanoparticles could minimize nonspecific adsorption of serum proteins (Wang et al., 2012; Zheng et al., 2013; Jiang et al., 2020b).

The performance of the SERS analyzer was compared with the paper-based lateral flow assay (LFA) (Figure 3C). The paper-based LFA is a standard method for antibody screening and had comparable LoD with the enzyme-linked immunosorbent assay (ELISA) but has the advantage of not relying on instruments. Herein, it was prepared using a standard nitrocellulose filter membrane (Quesada-González and Merkoçi, 2015), the testing line (T-line) was imprinted with anti-*IgG* antibody (goat anti-mouse), and the control line (C-line) was imprinted with rabbit *IgG* antibody (IBL, JP17312). To perform an *IgG* antibody test, a mixture containing three components was added: (1) gold nanoparticle solution with surface modified with S protein, (2) gold nanoparticle colloidal with surface modified with goat anti-rabbit *IgG* antibody (Abcam, ab6702), and (3) the target antibody (*IgG*). A gradual increase of color was observed when the concentration of *IgG* was varied between 0 and 66 nM, with an LoD found to be in between 4 and 8 nM. The results indicated that the magnetic liquid-SERS analyzer has superior LoD than paper-based LFA.

**FIGURE 4:**
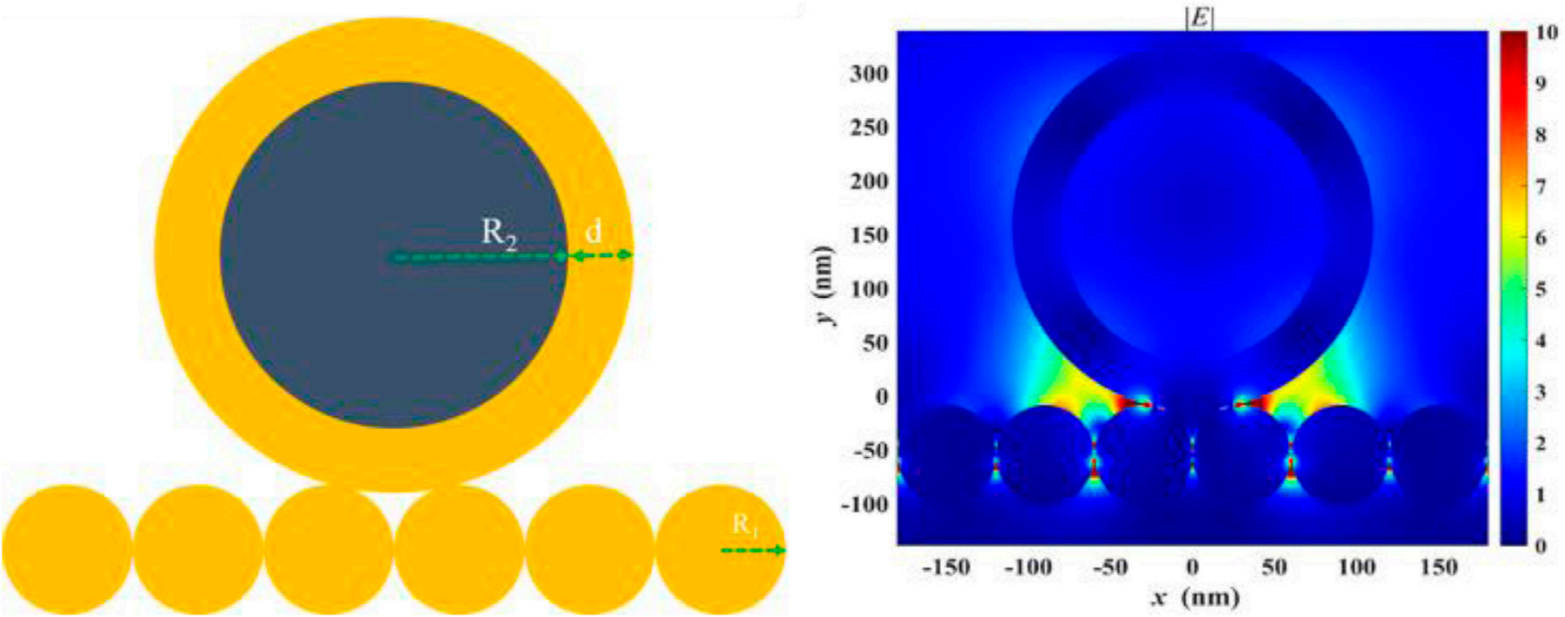
Finite-difference time-domain (FDTD) analysis showing enhanced electromagnetic (EM) field in between the gap formed by the Au@MNP and the liquid-state SERS substrate.

We attribute the excellent performance of the magnetically enhanced liquid state SERS to two reasons. First, sampling is improved when targets are enriched near the surface of the liquid–liquid interface of the plasmonic substrate. Second, magnetic enhancement leads to the formation of SERS hotspots between the substrate and the nanoparticles, in which gap-mode SERS can occur, as seen in Figure 4. In these scenarios, the EM enhancement factor can reach ∼10^8^ times or higher, which corresponds to ∼10^12^ time SERS enhancement, much higher than that of using the nanoparticle or substrate only. This is because

$$G_{SERS}\approx {(|E_{local}\left(\omega_{r}\right)|)}^{4}/{(|E_{0}\left(\omega_{0}\right)|)}^{4},$$

where *G* is the enhancement factor and *E* is the intensity of EM field at particular frequency (*ω*). The gap-mode system shows stronger enhancement than what can be achieved with the nanoparticles or substrate only, regardless of the magnetic core.2 Saying this, due to the limited scope of this work, a detailed analysis as to what extent each factor contribute to the final LoD is not done, but can be investigated in future studies. It is expected that with improved geometry of the Au@MNP, such as using star-shaped nanoparticles, the performance of the magnetic liquid SERS analyzer can be further improved. Another direction worth exploring is the effect of surface-enhanced resonant Raman scattering (SERRS) effects in this system. Resonant Raman can be important for its high-bond selectivity and capability for multiplexed detection (Faulds et al., 2005) This is particularly attractive when performed in ultraviolet waveband because DNAs and proteins absorb UV light strongly (Renard et al., 2019; Wu et al., 2021)

Since there are many studies in the literature focusing on ultrasensitive SERS measurements, a main advantage of this study is not to show the sensitivity is more superior than that of others but to demonstrate accessibility and high sensitivity can be achieved at the same time with ease with cost-effective instruments. In fact, pM sensitivity already answers to clinical demand, and the method is scalable at an industrial level. A comparison to several selected methods is shown in Table 1.

**TABLE 1:** Comparison of this work with typical SERS assay in the literature.

Assay type	Limit of detection	Scalability/accessibility	Time of operation	Instrument cost
Magnetic liquid SERS (this work)	10^–12^ M	Excellent	< 1 min	Low
				< 10 k USD
Liquid SERS (Ma et al., 2016; Tian et al., 2018)	10^–9^ M	Excellent	< 1 min	Low
				< 10 k USD
Nano star SERS (Indrasekara et al., 2014; Li et al., 2021)	10^–15^ M	Good	∼ 5 min	Varies
Single-molecule SERS (Yang et al., 2016; Li et al., 2021)	10^–18^ M	Poor	∼ 15 min	High
				Over 100 K USD

## Conclusions and Future Perspective

This study can be summarized as follows: (1) magnetic enhancements significantly improve the sensitivity of liquid-state SERS by lowering the limit of detection (LoD) by at least three orders of magnitude to enable pM-level detection of biomolecules. (2) The LoD for nucleic acid targets was determined by using DNA fragments associated with *Staphylococcus aureus*, a bacteria associated with cross-infections in hospitals. (3) Analysis for protein antibodies was determined with *IgG* antibodies used for COVID-19 screening, with a LoD of lower than 0.5 pM. The results were compared with paper-based LFA, showing significantly improved LoD of the magnetic liquid-SERS method. Due to the simple, reproducible process of substrate preparation and high performance of the assay toward both protein and nucleic acid targets, we expect this work to inspire further studies toward ultrasensitive SERS biosensors that could be scaled to mass production.

## Data Availability

The datasets presented in this study can be found in online repositories. The names of the repository/repositories and accession number(s) can be found in the article/Supplementary Material.

## References

[B1] Banholzer M. J. Millstone J. E. Qin L. Mirkin C. A. (2008). Rationally Designed Nanostructures for Surface-Enhanced Raman Spectroscopy. Chem. Soc. Rev. 37 (5), 885–897. 10.1039/b710915f 18443674 PMC8207723

[B2] Bell S. E. J. Charron G. Cortés E. Kneipp J. Chapelle M. L. Langer J. (2020). Towards Reliable and Quantitative Surface‐Enhanced Raman Scattering (SERS): From Key Parameters to Good Analytical Practice. Angew. Chem. Int. Ed. 59 (14), 5454–5462. 10.1002/anie.201908154 PMC7154527 31588641

[B3] Beveridge J. S. Stephens J. R. Williams M. E. (2011). The Use of Magnetic Nanoparticles in Analytical Chemistry. Annu. Rev. Anal. Chem. 4, 251–273. 10.1146/annurev-anchem-061010-114041 21417723

[B4] Chan C. Shahul S. Coleman C. Tesic V. Yeo K. J. A. J. o. C. P. (2020). Evaluation of the Truvian Easy Check COVID-19 IgM/IgG Lateral Flow Device for Rapid Anti-SARS-CoV-2 Antibody Detection. 10.1093/ajcp/aqaa221 PMC7665287 33135049

[B5] Chen P. Yin Z. Huang X. Wu S. Liedberg B. Zhang H. (2011). Assembly of Graphene Oxide and Au0.7Ag0.3 Alloy Nanoparticles on SiO2: A New Raman Substrate with Ultrahigh Signal-To-Background Ratio. J. Phys. Chem. C 115 (49), 24080–24084. 10.1021/jp208486m

[B6] Chen R. Du X. Cui Y. Zhang X. Ge Q. Dong J. (2020). Vertical Flow Assay for Inflammatory Biomarkers Based on Nanofluidic Channel Array and SERS Nanotags. Small 16 (32), e2002801. 10.1002/smll.202002801 32567225

[B7] Chen R. Cheng X. Zhang C. Wu H. Zhu H. He S. (2021). Sub-3 Nm Aluminum Nanocrystals Exhibiting Cluster-like Optical Properties. Small 17 (27), e2002524. 10.1002/smll.202170135 32812331

[B8] Chen Z. Zhang Z. Zhai X. Li Y. Lin L. Zhao H. (2020). Rapid and Sensitive Detection of Anti-SARS-CoV-2 IgG, Using Lanthanide-Doped Nanoparticles-Based Lateral Flow Immunoassay. Anal. Chem. 92 (10), 7226–7231. 10.1021/acs.analchem.0c00784 32323974

[B9] Ciallam D. März A. Böhme R. Theil F. Weber K. Schmitt M. (2012). Surface-enhanced Raman Spectroscopy: Concepts and Chemical Applications. Anal. Bioanal. Chem. 403, 27–54. 10.1007/s00216-011-5631-x 22205182

[B10] Deeks J. J. Dinnes J. Takwoingi Y. Davenport C. Spijker R. Taylor-Phillips S. (2020). Antibody Tests for Identification of Current and Past Infection with SARS-CoV-2. Cochrane Database Syst. Rev. 6 (6), CD013652. 10.1002/14651858.CD013652 32584464 PMC7387103

[B11] Ding S. Y. Yi J. Li J. F. Ren B. Wu D. Y. Panneerselvam R. (2016). Nanostructure-based Plasmon-Enhanced Raman Spectroscopy for Surface Analysis of Materials. Nat. Rev. Mater. 1 (6). 10.1038/natrevmats.2016.21

[B12] Du S. Su M. Jiang Y. Yu F. Xu Y. Lou X. (2019). Direct Discrimination of Edible Oil Type, Oxidation, and Adulteration by Liquid Interfacial Surface-Enhanced Raman Spectroscopy. ACS Sens. 4 (7), 1798–1805. 10.1021/acssensors.9b00354 31251024

[B13] Fan M. Andrade G. F. S. Brolo A. G. (2020). A Review on Recent Advances in the Applications of Surface-Enhanced Raman Scattering in Analytical Chemistry. Analytica Chim. Acta 1097, 1–29. 10.1016/j.aca.2019.11.049 31910948

[B14] Faulds K. Smith W. E. Graham D. (2005). DNA Detection by Surface Enhanced Resonance Raman Scattering (SERRS). Analyst 130 (8), 1125–1131. 10.1039/b500248f 16021211

[B15] Indrasekara A. S. D. S. Meyers S. Shubeita S. Feldman L. C. Gustafsson T. Fabris L. (2014). Gold Nanostar Substrates for SERS-Based Chemical Sensing in the Femtomolar Regime. Nanoscale 6 (15), 8891–8899. 10.1039/c4nr02513j 24961293

[B16] Jiang C. Hopfner F. Katsikoudi A. Hein R. Catli C. Evetts S. (2020). Serum Neuronal Exosomes Predict and Differentiate Parkinson's Disease from Atypical Parkinsonism. J. Neurol. Neurosurg. Psychiatry 91 (7), 720–729. 10.1136/jnnp-2019-322588 32273329 PMC7361010

[B17] Jiang C. Wang G. Hein R. Liu N. Luo X. Davis J. J. (2020). Antifouling Strategies for Selective *In Vitro* and *In Vivo* Sensing. Chem. Rev. 120 (8), 3852–3889. 10.1021/acs.chemrev.9b00739 32202761

[B18] Joseph V. Engelbrekt C. Zhang J. Gernert U. Ulstrup J. Kneipp J. (2012). Characterizing the Kinetics of Nanoparticle-Catalyzed Reactions by Surface-Enhanced Raman Scattering. Angew. Chem. Int. Ed. 51 (30), 7592–7596. 10.1002/anie.201203526 22806949

[B19] Kemp S. A. Collier D. A. Collier D. A. Datir R. P. Ferreira I. A. T. M. Gayed S. (2021). SARS-CoV-2 Evolution during Treatment of Chronic Infection. Nature 592 (7853), 277–282. 10.1038/s41586-021-03291-y 33545711 PMC7610568

[B20] Kneipp K. Kneipp H. Bohr H. G. (2006). Single-molecule SERS Spectroscopy. Top. Appl. Phys. 103, 261–277. 10.1007/3-540-33567-6_13

[B21] Kohmer N. Westhaus S. Rühl C. Ciesek S. Rabenau H. F. (2020). Clinical Performance of Different SARS‐CoV‐2 IgG Antibody Tests. J. Med. Virol. 92 (10), 2243–2247. 10.1002/jmv.26145 32510168 PMC7300776

[B22] Langer J. Jimenez de Aberasturi D. Aizpurua J. Alvarez-Puebla R. A. Auguié B. Baumberg J. J. (2020). Present and Future of Surface-Enhanced Raman Scattering. Acs Nano 14 (1), 28–117. 10.1021/acsnano.9b04224 31478375 PMC6990571

[B23] Li J. Gong X. Wang Z. Chen R. Li T. Zeng D. (2020). Clinical Features of Familial Clustering in Patients Infected with 2019 Novel Coronavirus in Wuhan, China. Virus. Res. 286, 198043. 10.1016/j.virusres.2020.198043 32502551 PMC7265838

[B24] Li J. R. Wuethrich A. Sina A. I. Cheng H. H. Wang Y. L. Behren A. (2021). A Digital Single-Molecule Nanopillar SERS Platform for Predicting and Monitoring Immune Toxicities in Immunotherapy. Nat. Commun. 12 (1). 10.1038/s41467-021-21431-w PMC7889912 33597530

[B25] Li M. Lin H. Paidi S. K. Mesyngier N. Preheim S. Barman I. (2020). A Fluorescence and Surface-Enhanced Raman Spectroscopic Dual-Modal Aptasensor for Sensitive Detection of Cyanotoxins. ACS Sens. 5 (5), 1419–1426. 10.1021/acssensors.0c00307 32314582 PMC7560972

[B26] Liu B. Liu J. (2019). Freezing-Driven DNA Adsorption on Gold Nanoparticles: Tolerating Extremely Low Salt Concentration but Requiring High DNA Concentration. Langmuir 35 (19), 6476–6482. 10.1021/acs.langmuir.9b00746 31008607

[B27] Liu B. Ni H. Zhang D. Wang D. Fu D. Chen H. (2017). Ultrasensitive Detection of Protein with Wide Linear Dynamic Range Based on Core-Shell SERS Nanotags and Photonic Crystal Beads. ACS Sens. 2 (7), 1035–1043. 10.1021/acssensors.7b00310 28750518

[B28] Long Y.-T. Gooding J. J. (2016). Surface-Enhanced Raman Spectroscopy for Sensing-Addressing a Real Challenge in Application. ACS Sens. 1 (8), 963. 10.1021/acssensors.6b00472

[B29] Ma Y. Liu H. Mao M. Meng J. Yang L. Liu J. (2016). Surface-Enhanced Raman Spectroscopy on Liquid Interfacial Nanoparticle Arrays for Multiplex Detecting Drugs in Urine. Anal. Chem. 88 (16), 8145–8151. 10.1021/acs.analchem.6b01884 27401135

[B30] Mattioli I. A. Hassan A. Oliveira O. N. Crespilho F. N. (2020). On the Challenges for the Diagnosis of SARS-CoV-2 Based on a Review of Current Methodologies. ACS Sens. 5 (12), 3655–3677. 10.1021/acssensors.0c01382 33267587

[B31] Pazos-Perez N. Pazos E. Catala C. Mir-Simon B. Gómez-de Pedro S. Sagales J. (2016). Ultrasensitive Multiplex Optical Quantification of Bacteria in Large Samples of Biofluids. Sci. Rep. 6, 29014. 10.1038/srep29014 27364357 PMC4929498

[B32] Quesada-González D. Merkoçi A. (2015). Nanoparticle-based Lateral Flow Biosensors. Biosens. Bioelectron. 73, 47–63. 10.1016/j.bios.2015.05.050 26043315

[B33] Renard D. Tian S. Ahmadivand A. DeSantis C. J. Clark B. D. Nordlander P. (2019). Polydopamine-Stabilized Aluminum Nanocrystals: Aqueous Stability and Benzo[a]pyrene Detection. Acs Nano 13 (3), 3117–3124. 10.1021/acsnano.8b08445 30807101

[B34] Sitjar J. Liao J.-D. Lee H. Tsai H.-P. Wang J.-R. Liu P.-Y. (2021). Bioelectronics, Challenges of SERS Technology as a Non-nucleic Acid or -antigen Detection Method for SARS-CoV-2 Virus and its Variants. Biosens. Bioelectron. 181, 113153. 10.1016/j.bios.2021.113153 33761416 PMC7939978

[B35] Su M. Li X. Zhang S. Yu F. Tian L. Jiang Y. (2019). Self-Healing Plasmonic Metal Liquid as a Quantitative Surface-Enhanced Raman Scattering Analyzer in Two-liquid-phase Systems. Anal. Chem. 91 (3), 2288–2295. 10.1021/acs.analchem.8b04893 30615424

[B36] Thai T. Zheng Y. Ng S. H. Mudie S. Altissimo M. Bach U. (2012). Self-Assembly of Vertically Aligned Gold Nanorod Arrays on Patterned Substrates. Angew. Chem. Int. Ed. 51 (35), 8732–8735. 10.1002/anie.201204609 22847940

[B37] Tian L. Su M. Yu F. Xu Y. Li X. Li L. (2018). Liquid-state Quantitative SERS Analyzer on Self-Ordered Metal Liquid-like Plasmonic Arrays. Nat. Commun. 9, 3642. 10.1038/s41467-018-05920-z 30194348 PMC6128918

[B38] Wang X. Wang C. Cheng L. Lee S.-T. Liu Z. (2012). Noble Metal Coated Single-Walled Carbon Nanotubes for Applications in Surface Enhanced Raman Scattering Imaging and Photothermal Therapy. J. Am. Chem. Soc. 134 (17), 7414–7422. 10.1021/ja300140c 22486413

[B39] Wei L. Chen Z. Shi L. Long R. Anzalone A. V. Zhang L. (2017). Super-multiplex Vibrational Imaging. Nature 544 (7651), 465–470. 10.1038/nature22051 28424513 PMC5939925

[B40] Wu H. Cheng X. Y. Dong H. G. Xie S. J. He S. L. (2021). Aluminum Nanocrystals Evolving from Cluster to Metallic State: Size Tunability and Spectral Evidence. Nano Res. 10.1007/s12274-021-3486-9

[B41] Wu Y. Tilley R. D. Gooding J. J. (2019). Challenges and Solutions in Developing Ultrasensitive Biosensors. J. Am. Chem. Soc. 141 (3), 1162–1170. 10.1021/jacs.8b09397 30463401

[B42] Yang S. Dai X. Stogin B. B. Wong T.-S. (2016). Ultrasensitive Surface-Enhanced Raman Scattering Detection in Common Fluids. Proc. Natl. Acad. Sci. USA 113 (2), 268–273. 10.1073/pnas.1518980113 26719413 PMC4720322

[B43] Yao H. Song Y. Chen Y. Wu N. Xu J. Sun C. (2020). Molecular Architecture of the SARS-CoV-2 Virus. Cell 183 (3), 730–738. 10.1016/j.cell.2020.09.018 32979942 PMC7474903

[B44] Ye Y. Liu Z. C. Zhang W. Y. Cheng X. Y. He S. L. (2019). Freeze-Facilitated Ligand Binding to Plasmonic Gold Nanorods. Adv. Mater. Inter. 6 (23), 1900975. 10.1002/admi.201900975

[B45] Ying F. Seong N. H. Dlott D. D. J. S. (2008). Measurement of the Distribution of Site Enhancements in Surface-Enhanced Raman Scattering. Science **,** 321 (5887), 388–392. 10.1126/science.1159499 18583578

[B46] Zhang D. Huang L. Liu B. Su E. Chen H.-Y. Gu Z. (2018). Quantitative Detection of Multiplex Cardiac Biomarkers with Encoded SERS Nanotags on a Single T Line in Lateral Flow Assay. Sensors Actuators B: Chem. 277, 502–509. 10.1016/j.snb.2018.09.044

[B47] Zheng Y. Soeriyadi A. H. Rosa L. Ng S. H. Bach U. Justin Gooding J. (2015). Reversible Gating of Smart Plasmonic Molecular Traps Using Thermoresponsive Polymers for Single-Molecule Detection. Nat. Commun. 6, 8797. 10.1038/ncomms9797 26549539 PMC4667617

[B48] Zheng Y. Thai T. Reineck P. Qiu L. Guo Y. Bach U. (2013). DNA-directed Self-Assembly of Core-Satellite Plasmonic Nanostructures: A Highly Sensitive and Reproducible Near-IR SERS Sensor. Adv. Funct. Mater. 23 (12), 1519–1526. 10.1002/adfm.201202073

