# Supplementary material for: Magnetically Enhanced Liquid SERS for Ultrasensitive Analysis of Bacterial and SARS-CoV-2 Biomarkers
Source: Front Bioeng Biotechnol. 2021 Sep 29;9:735711. doi: 10.3389/fbioe.2021.735711 (PMC8511622; doi:10.3389/fbioe.2021.735711)
Supplement: Supplementary file 1 [file DataSheet1.PDF]

Magnetically Enhanced Liquid SERS for Ultrasensitive Analysis of Bacterial and SARS-CoV-2 Biomarkers

Zhang Ji,[1] Chuan Zhang, [1] Yang Ye, [1,2,3], Jiali Ji, [1] Hongguang Dong,[1] Erik Forsberg,[1]

Xiaoyu Cheng,[1,2,3*] Sailing He[1,2,3*]

1. National Engineering Research Center for Optical Instruments, College of Optical Science and Engineering, Zhejiang University, Hangzhou, 310052, China
2. Ningbo Research Institute, Ningbo, 310050, China
3. ZJU-TU/e Joint Research Institute of Design, Optoelectronic and Sensing

Table of contents

Content	Page No
1. Methods and Materials	2
1.1 Chemicals	2
1.2 Preparation of Gold Coated Magnetic Nanoparticles	3
1.3 Surface Modification of Au@MNPs	4
1.4 Preparation of Liquid SERS Substrate	4
1.5 SERS Measurements	4
1.6 Preparation of Paper Based Lateral Flow Sensors	4
1.7 Finite-Difference Time-Domain Simulations	5
2. Additional Results and Discussions	6
2.1 Characterization of Au@MNP	6
2.2 Characterization of Plasmonic Liquid SERS Substrate	7
2.3 SERS Results without Substrate	8
3. References	9

1. Methods and Materials

1.1 Chemicals

Iron (III) chloride hexa-hydrate ($FeCl_3 \cdot 6H2O$), manganese (II) chloride tetra-hydrate ($MnCl_2$), ethylene glycol (EG), diethylene glycol (DEG), polyethylene glycol (PEG) absolute ethanol (EtOH) and sodium acetate (CH_3COONa) were purchased from Sinopharm Chemical Reagent Co., Ltd. (China). Polyvinylpyrrolidone (40 K, PVP) was purchased from TCI; polyethyleneimine (PEI) and chloroauric acid ($HAuCl_4$) from Sigma; hydroxylamine hydrochloride ($NH_4OH \cdot HCl$) and sodium citrate ($Na_3C_6H_5O_7$) from Aladdin; and sodium borohydride ($NaBH_4$) from Fluka. Nucleic acids, the sequence information of which is shown as Table 1, were purchased from Sangon Biotech; antibodies (Cat No 0018) and S 2 proteins (Cat No HEK293) from Native Antigen; and whole blood mimic from Solarbio biopharmaceuticals (Cat No A7930).

Table 1. Oligonucleotides Used in This Study

Oligo Name	Length(bp)	Sequence (5' to 3')	Modification
Target Strand	40	CTTCAGAACCACTTCTATTT ACGCCATTATCTGTTTGTGA	
S-signal-Cy3	20	TCACAAACAGATAATGGCGT-Cy3	5'Cy3
S-capture-SH	30	AAATAGAAGTGGTTCAAG-PEG-A10SH	3'SH-C6

1.2 Preparation of Gold-Coated Magnetic Nanoparticles

$MnFe_2O_4$ magnetic cores were synthesized using previously published protocols.[1-3] Briefly: 360 mg of $FeCl_3 \cdot 6H_2O$ (1.33 mM) and 131.94 mg of $MnCl_2$ are added to a 20 mL mixture containing 10 mL diethylene glycol (DEG) and 10 mL ethylene glycol (EG), and stirred for 20 min until the solution became transparent. 2 g of PVP powder was added and the sample is heated at 120ºC, until the PVP powder was completely dissolved. After heating, 1.5 g of sodium acetate were magnetically stirred for 30 minutes. The solution was reacted at 205 ºC for 10 h. The synthesized magnetic nanoparticles were collected using a

magnet and the sample was washed five times with ethanol, and vacuum dried for 24 hours. The final product was obtained as a dark powder.

Gold coated magnetic nanoparticles were prepared by synthesizing gold seeds then attached to the magnetic core to enable shell growth. To produce gold seeds of ~4 nm in size, $HAuCl_4$ solution (25 mM, 2.22 mL) and sodium citrate solution (5 mM, 11.11 mL) are added to 200 mL of dH_2O. The mixture was stirred at 900 rpm for 5 minutes, after which 5.56 mL of freshly prepared $NaBH_4$ solution (100 mM) was quickly added. The mixture was stirred for 4 hours and then stored at 4°C for later use.

To coat the $MnFe_2O_4$ nanoparticles with Au seeds, 1 g of polyetherimide (PEI) polymer was added to 200 mL of diH_2O to prepare a 5 mg/mL PEI solution. 100 mg of the previously prepared $MnFe_2O_4$ nanoparticle powder was added to the PEI solution then sonicated for 20 min. Following magnetic separation, the mixture was washed five times with dH2O. A gold seed solution (200 mL, 4 nm diameter) was then added, and the mixture sonicated for 30 min. Product was collected with a magnet and washed five times with dH2O. A solution with 1 mg/mL Au seeds -PEI@$MnFe_2O_4$ was then prepared. For shell growth, 5 mL of the Au seeds-PEI@$MnFe_2O_4$ solution was diluted four times with 15 mL of dH_2O. 250 mg PVP was added after the mixture was sonicated and the solid dissolved. 200 uL of $NH_4OH{\cdot}HCl$ solution (50 mg/mL) was added and sonicated for 3 minutes, after which a HAuCl4 solution (25mM) was added and the mixture further stirred for 5 min. Finally the prepared Au@MNPs were collected using a magnet, washed three times with deionized water, then dispersed in 5 mL of deionized water. The product was stored at 4 ºC.

1.3 Surface Modification of Au@MNP

Au@MNPs modified by nucleic acids were prepared using standard freeze-induced protocol.[4] A stock solution of single stranded DNA (ssDNA) was prepared at 100 μM. In a typical surface modification protocol, 4 μL of stock DNA was pre-treated with TCEP (10 mM), then purified with Centri-Spin

purification columns (CS200 Princeton Separations) before added to 100 μL of Au@MNP solution (5 mg/mL).

Au@MNPs modified by proteins were prepared with step-by-step self-assembled process. 500 μL Au@MNPs (5 mg/mL) was mixed with 20 mg SH-PEG3400-biotin. The mixture was sonicated for 3 minutes to disperse the particles uniformly, and incubated overnight at room temperature, after which it was washed three times with 1*PBS to remove free PEG molecules. For protein conjugation, a streptavidin-labelled S2 protein was prepared using kit produced by Abcam (Cat No ab102921). 10 μL of S2 protein solution (10 mg/mL) was added to Au@MNP and the mixture incubated at 37°C for 20 minutes. Antibody modified Au@MNPs were purified by magnetic separation.

1.4 Preparation of liquid SERS substrate

In a typical experiment, 3 mL of 60 nm-sized gold nanoparticle sol (0.06 nM) and 1 mL of chloroform were added into a hydrophilized glass sample tube. The mixture was vigorously shaken for 30 seconds until a film with visible metallicity was observed at the interface between the two immiscible solvents.

1.5 SERS measurements

SERS measurements were performed using a home-built system based on commercially available components. The Raman system was composed of a Bitatech Kraman spectrometer (BTC162E-785H) and a 785 nm continuous wave (CW) laser connected with a fibre probe (InPhotonics). The excitation light power was adjusted to 100 mW with an integration time of 15 seconds, when the diameter of the focusing spot was ~100 μm. The same sample was measured six times to produce a final spectrum for each measurement.

1.6 Preparation of Paper Based Lateral Flow Sensors

Paper based lateral-flow sensors were prepared using nitrocellulose filter membrane, the testing line (T-line) was imprinted with goat anti-mouse *IgG* antibody, and the control line (C-line) was imprinted with rabbit *IgG* antibody.

To perform the test, a mixture containing three components were added: gold nanoparticle colloid with surface modified by S protein; gold nanoparticle colloidal with surface modified by goat anti-rabbit *IgG* antibody; the target antibody (*IgG*).

1.7 Finite-Difference Time-Domain Simulations

Numerical simulations were performed using the finite-difference time-domain technique. The radius of the Fe_3O_4 core is 80 nm and the thickness of the Au shell was 30 nm. The diameter of self-assembled gold nanoparticles is 60 nm. To simplify the model, a two-dimensional simulation area was used. The whole sample was immersed in a liquid environment with a background refractive index of 1.446. A plane wave is incident vertically into the sample along the negative Y-axis. The Periodic boundary conditions are applied in the X direction and PML boundary conditions are applied in the y direction. The mesh size was set to 0.25 nm.

2. Additional Results and Discussions

2.1 Characterization of Au@MNPs

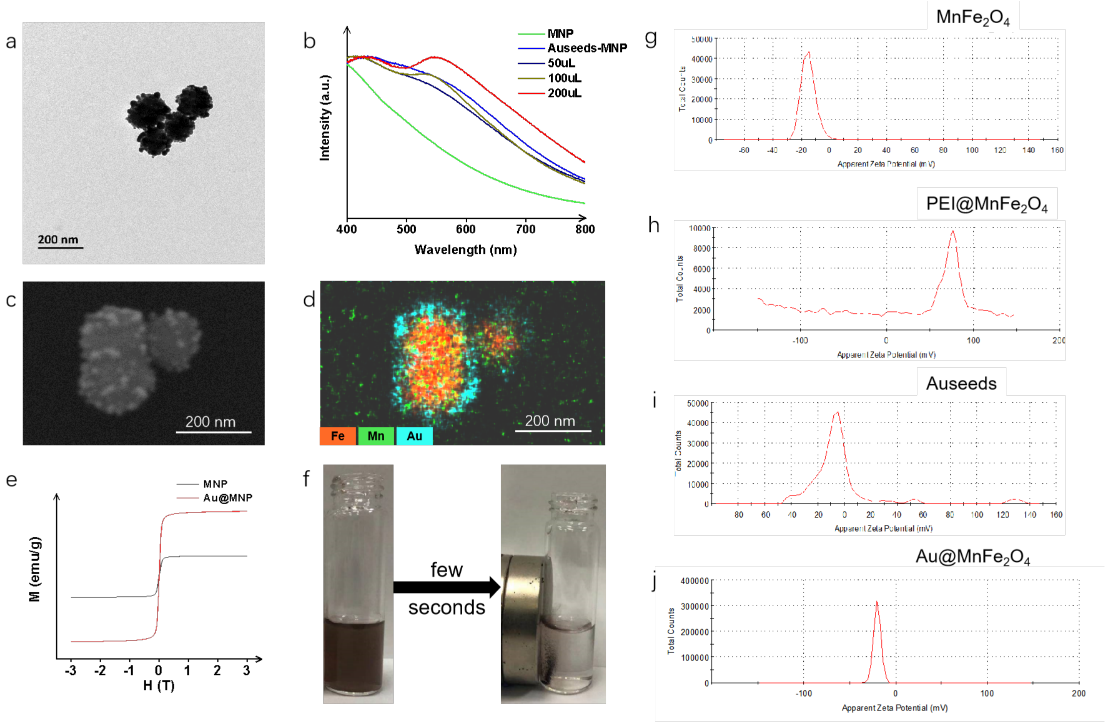

Figure S. 1 Characterizations of the Au@MNPs used in this study. (a) Typical TEM images of the synthesized nanoparticles. (b) UV-visible absorption spectra of the synthesized nanoparticles. (c) Scanning Electron Microscopy (SEM) of a typical Au@MNP, and (d) the corresponding energy dispersive spectrometry (EDS) mapping. (e) Magnetic hysteresis loop, and (f) a photo demonstrating response of the Au@MNPs when applied with an external magnetic field. (g) – (j) Zeta potential measurement showing varied surface charge during the synthesis showing varied charge of the surface.

Gold coated magnetic nanoparticles (Au@MNPs) were synthesized using a established protocol.[1-3, 5] Briefly summarized, Au@MNPs were prepared by synthesizing a magnetic core, which was sequentially coated with a layer of polyetherimide (PEI) polymer and then a gold shell. The material properties of the Au@MNPs were first characterized and the results are shown in Figure S. 1.

2.2 Characterization of Liquid SERS Interface

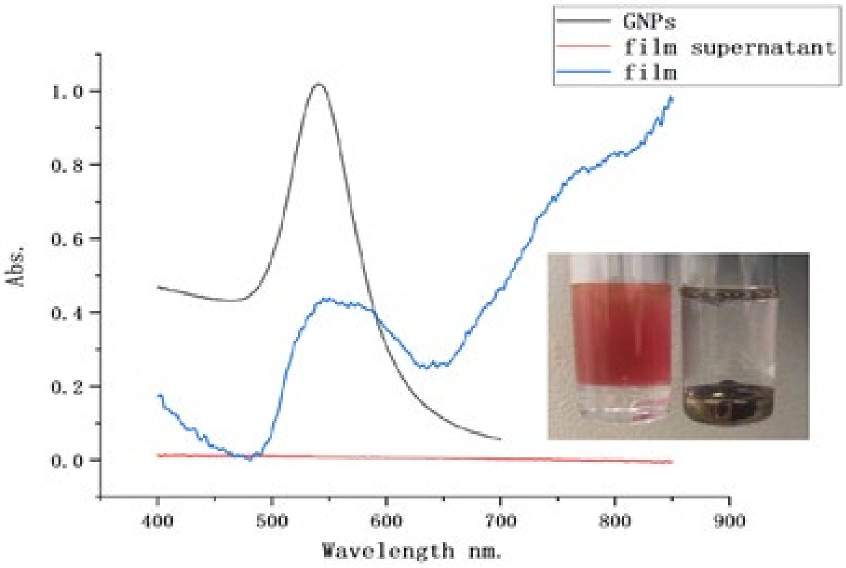

Figure S. 2 Absorption spectra of gold nanoparticles before/after forming the liquid-state SERS substrate. Inset shows a picture of the substrate in vial.

Direct assembly of the nanoparticles were driven by interfacial *Van der Waals* interactions in the gold colloid at the surface of the two immiscible solvents (water and chloroform). This produced a self-healing film with metallicity, and the fabrication process could be finished in ~1 min with high reproducibility. Absorption measurements revealed a clear plasmon band at ~548 nm, which was slightly red-shifted compared to colloidal nanoparticles, and a broad shoulder band was seen in the 650 nm - 900 nm range (Figure S.5), which agreed with observations in the literature.[6]

2.3 SERS Results without the Substrate

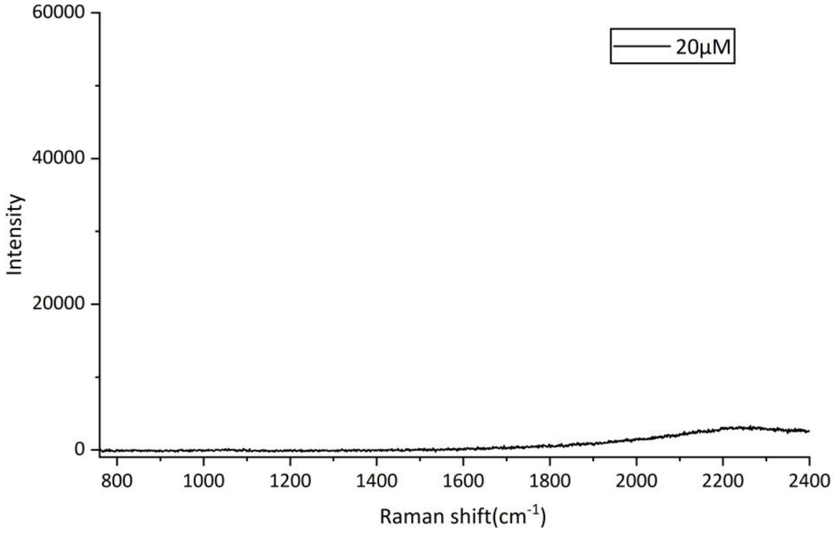

Figure S. 3 Ultrasensitive detection of nucleic acid fragments of S. aureus without liquid-state SERS platform. No Raman signal could be detected even with a high concentration of probe (20 μM) was added.

The method constructed in this experiment (without liquid-state SERS platform) was used for SERS analysis of high concentration S. aureus, as shown in Figure S. 3 Ultrasensitive detection of nucleic acid fragments of S. aureus without liquid-state SERS platform. No Raman signal could be detected even with a high concentration of probe (20 μM) was added.. The results show that no characteristic peaks are obtained. Therefore, the liquid-state SERS substrate is essential for generating the high performance analysis in this work.

3. References

1. Goon, I. Y.; Lai, L. M. H.; Lim, M.; Amal, R.; Gooding, J. J., 'Dispersible electrodes': a solution to slow response times of sensitive sensors. *Chem Commun* **2010,** *46* (46), 8821-8823.

2. Xuan, S. H.; Wang, F.; Wang, Y. X. J.; Yu, J. C.; Leung, K. C. F., Facile synthesis of size-controllable monodispersed ferrite nanospheres. *J Mater Chem* **2010,** *20* (24), 5086-5094.

3. Chuah, K.; Lai, L. M. H.; Goon, I. Y.; Parker, S. G.; Amal, R.; Gooding, J. J., Ultrasensitive electrochemical detection of prostate-specific antigen (PSA) using gold-coated magnetic nanoparticles as 'dispersible electrodes'. *Chem Commun* **2012,** *48* (29), 3503-3505.

4. Liu, B. W.; Liu, J. W., Freezing-Driven DNA Adsorption on Gold Nanoparticles: Tolerating Extremely Low Salt Concentration but Requiring High DNA Concentration. *Langmuir* **2019,** *35* (19), 6476-6482.

5. Gloag, L.; Mehdipour, M.; Chen, D. F.; Tilley, R. D.; Gooding, J. J., Advances in the Application of Magnetic Nanoparticles for Sensing. *Advanced Materials* **2019,** *31* (48).

6. Pahlow, S.; Meisel, S.; Cialla-May, D.; Weber, K.; Rosch, P.; Popp, J., Isolation and identification of bacteria by means of Raman spectroscopy. *Adv Drug Deliver Rev* **2015,** *89*, 105-120.
